# Assessing the potential of subcutaneous biologging tools for physiological monitoring in a large terrestrial mammal, the Guanaco (*Lama guanicoe*)

**DOI:** 10.1093/conphys/coag048

**Published:** 2026-07-15

**Authors:** Antonella Panebianco, María V Rago, Pablo F Gregorio, Fiama Peña Lodis, Megan A Owen, Valeria Pomponio, María P Pompei, Melina Anello, Natalia M Schroeder, Ramiro Ovejero, Pablo Carmanchahi

**Affiliations:** Grupo de Investigación de Eco-Fisiología de Fauna Silvestre (GIEFAS), Instituto de Investigaciones en Biodiversidad y Medio Ambiente, Consejo Nacional de Investigaciones Científicas y Técnicas, Centro Regional Universitario San Martín de los Andes, Universidad Nacional del Comahue, Pasaje de la Paz 235, CP 8370, San Martín de los Andes, Neuquén, Argentina; Instituto de Investigaciones en Biodiversidad y Medio Ambiente, Consejo Nacional de Investigaciones Científicas y Técnicas, Sede Junín de los Andes, Ruta N° 61 km 3, CP 8371, Junín de los Andes, Neuquén, Argentina; Grupo de Investigación de Eco-Fisiología de Fauna Silvestre (GIEFAS), Instituto de Investigaciones en Biodiversidad y Medio Ambiente, Consejo Nacional de Investigaciones Científicas y Técnicas, Centro Regional Universitario San Martín de los Andes, Universidad Nacional del Comahue, Pasaje de la Paz 235, CP 8370, San Martín de los Andes, Neuquén, Argentina; Grupo de Investigación de Eco-Fisiología de Fauna Silvestre (GIEFAS), Instituto de Investigaciones en Biodiversidad y Medio Ambiente, Consejo Nacional de Investigaciones Científicas y Técnicas, Centro Regional Universitario San Martín de los Andes, Universidad Nacional del Comahue, Pasaje de la Paz 235, CP 8370, San Martín de los Andes, Neuquén, Argentina; Witral, Red de Investigaciones en Conservación y Manejo de Vida Silvestre en Sistemas Socio-ecológicos, Instituto Argentino de Investigaciones de las Zonas Áridas, Consejo Nacional de Investigaciones Científicas y Técnicas, Av. Ruiz Leal s/n, Parque General San Martín, CP 5500, Mendoza, Argentina; San Diego Zoo Wildlife Alliance, 15600 San Pasqual Valley Rd., CP 92027, Escondido, CA, USA; Grupo de Investigación de Eco-Fisiología de Fauna Silvestre (GIEFAS), Instituto de Investigaciones en Biodiversidad y Medio Ambiente, Consejo Nacional de Investigaciones Científicas y Técnicas, Centro Regional Universitario San Martín de los Andes, Universidad Nacional del Comahue, Pasaje de la Paz 235, CP 8370, San Martín de los Andes, Neuquén, Argentina; Grupo de Investigación en Arqueología Andina, Consejo Nacional de Investigaciones Científicas y Técnicas, Universidad Nacional de Tucumán, Miguel Lillo 205, CP 4000, San Miguel de Tucumán, Tucumán, Argentina; Grupo de Investigación de Eco-Fisiología de Fauna Silvestre (GIEFAS), Instituto de Investigaciones en Biodiversidad y Medio Ambiente, Consejo Nacional de Investigaciones Científicas y Técnicas, Centro Regional Universitario San Martín de los Andes, Universidad Nacional del Comahue, Pasaje de la Paz 235, CP 8370, San Martín de los Andes, Neuquén, Argentina; Grupo de Investigación de Eco-Fisiología de Fauna Silvestre (GIEFAS), Instituto de Investigaciones en Biodiversidad y Medio Ambiente, Consejo Nacional de Investigaciones Científicas y Técnicas, Centro Regional Universitario San Martín de los Andes, Universidad Nacional del Comahue, Pasaje de la Paz 235, CP 8370, San Martín de los Andes, Neuquén, Argentina; Witral, Red de Investigaciones en Conservación y Manejo de Vida Silvestre en Sistemas Socio-ecológicos, Instituto Argentino de Investigaciones de las Zonas Áridas, Consejo Nacional de Investigaciones Científicas y Técnicas, Av. Ruiz Leal s/n, Parque General San Martín, CP 5500, Mendoza, Argentina; Facultad de Ciencias Agrarias, Universidad Nacional de Cuyo, Almirante Brown 500, CP M5528AHB, Luján de Cuyo, Mendoza, Argentina; Grupo de Investigación de Eco-Fisiología de Fauna Silvestre (GIEFAS), Instituto de Investigaciones en Biodiversidad y Medio Ambiente, Consejo Nacional de Investigaciones Científicas y Técnicas, Centro Regional Universitario San Martín de los Andes, Universidad Nacional del Comahue, Pasaje de la Paz 235, CP 8370, San Martín de los Andes, Neuquén, Argentina; Instituto de Biodiversidad Neotropical, IBN-CONICET-UNT; CCT NOA SUR, Cúpulas Horco Molle, CP 4000, Yerba Buena, Tucumán, Argentina; Grupo de Investigación de Eco-Fisiología de Fauna Silvestre (GIEFAS), Instituto de Investigaciones en Biodiversidad y Medio Ambiente, Consejo Nacional de Investigaciones Científicas y Técnicas, Centro Regional Universitario San Martín de los Andes, Universidad Nacional del Comahue, Pasaje de la Paz 235, CP 8370, San Martín de los Andes, Neuquén, Argentina

**Keywords:** Circadian rhythms, device retention, heart rate, *Lama guanicoe*, physiologging, subcutaneous body temperature

## Abstract

Physiological responses of animals to environmental and anthropogenic stressors are fundamental to effective conservation and wildlife management. Continuous monitoring of heart rate (HR) and body temperature yields key information on energy expenditure, stress, reproduction and health status. However, acquiring long-term physiological data from free-ranging large mammals remains technically challenging. Subcutaneous implantable biologgers represent a promising approach, yet concerns about device retention, data quality and animal welfare can constrain their widespread adoption. In this study, we assessed the feasibility of subcutaneous biologging to monitor HR and subcutaneous body temperature in guanacos (*Lama guanicoe*), the largest wild ungulate in South America and a species currently facing increasing anthropogenic threats. Two implantation trials were performed in captive adult females, each employing distinct biologger models and surgical techniques. The initial trial, which used a larger device implanted in the pectoral region, resulted in complete device expulsion within 4 weeks, despite successful surgeries and apparent normal wound healing. Conversely, the second trial, utilizing a smaller logger implanted in the intercostal region, achieved full device retention throughout the deployment period, with minimal tissue reaction and successful data retrieval. While the results suggest that device size and implantation site are influential, the simultaneous variation in multiple aspects of experimental design prevents a definitive attribution of retention success to a single variable. The retained devices provided the first continuous records of HR and subcutaneous body temperature in this species, revealing pronounced daily rhythms and temperature patterns that correlated with ambient temperature. Overall, these results underscore the necessity of species-specific pilot validation before field deployment to minimize study failure and ensure animal welfare. By outlining practical considerations for implantable biologging in large ungulates, this study provides insight to help guide the development of physiological monitoring protocols for future field applications.

## Introduction

Physiological monitoring plays a central role in understanding how animals respond to environmental and anthropogenic stressors in natural habitats (Cooke *et al.*, 2014; Bergman *et al.*, 2019). Physiological indicators, such as heart rate (HR) and body temperature, serve as reliable proxies for metabolic rate, as they are closely linked to oxygen consumption and metabolic heat production, respectively, providing important indicators of an individual’s allostatic load and welfare state (Moen, 1978; Green, 2011; Rey *et al.*, 2015). In conservation physiology, these metrics are widely used to detect disease and stress (Ovejero *et al.*, 2016; Beaulieu & Masilkova, 2024), identify reproductive events such as gestation and parturition (Friebe *et al.*, 2014; Marozzi *et al.*, 2024), assess responses to anthropogenic disturbances (Ditmer *et al.*, 2015) and develop mechanistic bioenergetic models that link individual metabolic states to population dynamics and survival (Kirchner, 2024).

Historically, collecting fine-scale, long-term physiological data in free-ranging mammals has posed significant methodological challenges. Traditional monitoring approaches are often restricted to short observation periods or discrete temporal ‘snapshots,’ limiting their ability to capture the full dynamics of animal responses over time (Wassmer *et al.*, 2020). Recent advances in biologging technology offer a promising alternative through the use of subcutaneous biologgers, which enable continuous, remote monitoring of physiological variables while reducing the need for repeated handling or prolonged human presence, which typically influence stress and behaviour in wildlife (Beaulieu & Masilkova, 2024). Subcutaneous biologgers have been applied across a range of taxa, including sheep (*Ovis aries*, Fuchs *et al.*, 2019), moose (*Alces alces*, Kirchner, 2024), reindeer (*Rangifer tarandus platyrhynchus,*  Trondrud *et al.*, 2021), several bear species (*Ursus americanus*, *Ursus arctos*, Laske *et al.*, 2018) and fishes (*Gadus morhua* L., Bjarnason *et al.*, 2019; *Salmo salar*, Zrini & Gamperl, 2021; *Thunnus thynnus*, Rouyer *et al.*, 2023), providing high-resolution measurements of HR and internal body temperature over extended periods. Furthermore, biologgers can be useful for quantifying physiological responses caused by other monitoring technologies, such as unoccupied aerial vehicles (i.e. drones), which have been shown to elicit HR increases in some species despite the animals showing little to no visible behavioural reaction (Ditmer *et al.*, 2015; Weimerskirch *et al.*, 2018).

Despite their increasing use, subcutaneous biologgers present unresolved challenges related to implantation procedures, data quality, device performance and animal welfare, particularly in large, free-ranging mammals (Hawkes *et al.*, 2021). Many studies provide limited methodological detail on device deployment, validation, handling and post-implantation monitoring, which constrains reproducibility and hinders the robust assessment of device impacts under field conditions (Williams *et al.*, 2020; Beaulieu & Masilkova, 2024; Payne *et al.*, 2026). Particularly, field validation of physiological signals obtained from subcutaneous biologgers remains limited, partially because the lack of direct observation and experimental control in the wild prevents independent validation of sensor outputs and disentangling device-related effects from environmental influences (Beaulieu & Masilkova, 2024). Accordingly, evaluations in controlled or semi-controlled environments are a desirable first step, as they permit the systematic development of species-specific baseline data and the validation of device functionality before broad application in free-ranging populations (Leimgruber *et al.*, 2023).

Beyond signal validation and deployment protocols, device retention remains a critical methodological challenge. Although subcutaneous biologgers have been successfully applied across taxa, implant loss or rejection can occur despite appropriate surgical technique and post-operative care. Evidence from large mammals and other species indicates that foreign body responses, device characteristics and implantation site may interact in species-specific ways that are difficult to predict *a priori* (Laske *et al.*, 2011; Mayer *et al.*, 2022). Because expulsion may occur weeks following implantation and is not always externally evident, it constitutes a potential point of study failure, with implications for data continuity, statistical power and animal welfare (Williams *et al.*, 2020; Beaulieu & Masilkova, 2024). These uncertainties reinforce the importance of stepwise validation of biologgers in captive or semi-controlled settings—even with small sample sizes—to evaluate wound healing, retention dynamics and device performance before field deployment (Forin-Wiart *et al.*, 2019; Leimgruber *et al.*, 2023; Beltran *et al.*, 2024).

In this study, we used the guanaco (*Lama guanicoe*) as a model species to evaluate subcutaneous physiological biologging in a wild large herbivore. Guanacos are the largest native ungulates inhabiting South American arid lands and are facing increasing anthropogenic threats (Franklin, 1982; Carmanchahi *et al.,* 2022a). Guanaco populations have declined substantially since the 1800s, and their current distribution is highly heterogeneous. Ongoing threats include habitat degradation due to overgrazing, competition with introduced herbivores and unsustainable hunting and poaching, among others (Baldi *et al.*, 2016; Carmanchahi *et al.*, 2022a). Here, we describe our experience with two biologger models that showed markedly different implantation success. Through a detailed description of implantation procedures and post-implantation monitoring, we synthesize methodological insights and key considerations regarding device model and performance to provide practical guidance for the application of subcutaneous biologging in guanacos and other large ungulates. We also present and validate the physiological data obtained, showing their reliability for use in conservation physiology studies. Finally, we examine daily patterns of subcutaneous body temperature and their relationship with ambient temperature to assess how guanacos adjust thermoregulation under natural environmental conditions.

## Materials and Methods

### Ethical statement

The experimental methodology described here was evaluated and approved by the CICUAL (Institutional Committee for the Care and Use of Laboratory or Experimental Animals) of INIBIOMA-CONICET-UNCo, Argentina, under protocol No. 3/2019. The research was also approved by the Secretary of Territorial Development and Environment (Disp. 002/20) of the Neuquén province (Argentina).

### Study site and animals

This study was performed at the ‘Los Peucos’ farm (Neuquén province, Argentina; 39.73° S, 71.06° W), located in the Patagonian steppe (altitude ~1100 masl). The mean annual temperature is 10°C, with a mean annual precipitation of 800 mm, concentrated in winter (León *et al.*, 1998; Bran *et al.*, 2002). The vegetation is characterized by a gramineous steppe and by native forest and exotic pine (ponderosa pine*, Pinus ponderosa,* Douglas ex P. Lawson & C. Lawson) plantations. This farm holds about 400 guanacos living in captive conditions in ~5000 ha paddocks with access to natural pasture and water, and they are annually enclosed and sheared for fibre production. For both trials, we captured adult non-pregnant females (between 5 and 7 years old, ~100 kg) for biologger deployment.

### Implantation methods

#### Trial 1

Before surgery, loggers were sterilized with glutaraldehyde (2%) for a minimum of 10 h (Star Oddi, 2019). All surgeries were conducted inside a barn by the same veterinarian (M.V. Rago), following animal welfare protocols (Carmanchahi *et al.*, 2022b). On a clean table, we prepared medicines, equipment and sterilized surgical kits for each animal. Just before surgery, the guanacos were captured, blindfolded, placed in right lateral recumbency and held on a gurney. The surgical area (around 8″ × 8″) was shaved and scrubbed with a 10% povidone iodine solution. After surgery, guanacos were identified with a coloured collar to facilitate recognition from a distance and released to an adjacent pen for further monitoring. We assessed body condition (BCI) on the day of implantation and again at logger retrieval by palpating the degree of sharpness of spinous processes, muscle mass and fat cover adjacent to the lumbar vertebrae (Audige *et al.*, 1998; Taraborelli *et al.*, 2017). BCI ranges from 1 (very poor condition, cachexia) to 5 (very good condition, fat).

Trial 1 was initially designed to conduct a drone disturbance experiment to assess physiological and behavioural responses of guanacos to drone approach; however, we do not report details specific to that study in this manuscript. We fitted three guanacos with DST centi-HRT ACT loggers (15-mm diameter × 46-mm length, 19 g, Star-Oddi, Iceland) between March and May 2022 (Table 1). These devices monitor HR via a single-channel electrocardiogram (ECG) amplifier using two integrated electrodes. HR values were derived from mean R–R intervals obtained from ECG burst measurements (Bjarnason *et al.*, 2019). Bursts were graded with a quality index (QI, QI = 0 great quality, QI = 3 poor quality, Trondrud *et al.*, 2021; Star Oddi, 2024). Each logger also records subcutaneous body temperature (Tsc; resolution: 0.032°C; accuracy: ±0.2°C) and tri-axial acceleration (resolution: 2 mg). To optimize memory and battery performance, loggers were programmed using multiple recording intervals to register fine-scale data during the drone approach and low-frequency sampling otherwise (Supplementary Material  1).

**Table 1:** Comparison of surgical protocols, logger characteristics and retention outcomes for two subcutaneous biologger models implanted in guanacos (*L. guanicoe*)

	**Trial 1**	**Trial 2**	**Practical considerations**
Model	DST centi-HRT ACT	DST micro-HRT	
Size	15-mm diameter × 46-mm length	8.3-mm diameter × 25.4-mm length	
Sterilization	Glutaraldehyde (2%) for a minimum of 10 h		
Implantation site	Pre-sternal region on the left side of the pectoral area	Left intercostal region	
	Shaved and scrubbed with a 10% povidone iodine solution		
Anaesthesia	Local	General anaesthesia	
Surgical outcome	No surgical complications, limited haemorrhaging	No surgical complications, less haemorrhaging than trial 1	Successful implantation does not guarantee retention
Post-implantation monitoring	Every 48 h for a week	Every 48 h for 30 days	Intensive post-implantation monitoring beyond the initial 2 weeks may be unnecessary
Retention	100% expulsion ≤4 weeks	100% retention after 4 weeks	Retention should be empirically validated
Inferred tissue response	Evidence consistent with acute foreign body response	No acute foreign body response	Size and implantation site possibly associated with inflammatory response

All surgeries were conducted inside a barn by the same veterinarian following animal welfare protocols (Carmanchahi *et al.*, 2022b).

**Figure 1:**
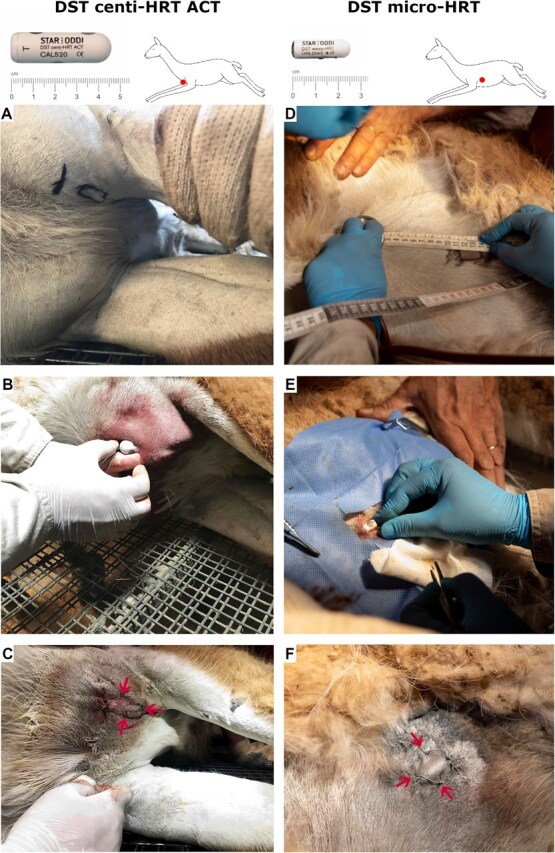
Orientation and placement of the loggers in guanacos. Dots in the guanaco silhouettes at the top of the figure indicate loggers’ implantation site. (A–C) DST centi-HRT ACT loggers were placed on the pre-sternal region on the left side of the pectoral area. (D–F) DST micro-HRT loggers were placed on the left intercostal region. Arrows in photos C and F indicate stitches around the logger to make a three-sided pocket. Logger photos with the scales are adapted from https://www.star-oddi.com/

Loggers were implanted subcutaneously on the pre-sternal region on the left side of the pectoral area, approximately 20 cm from the heart, following the manufacturer’s suggestion (Fig. 1A). This implantation site was identified by auscultation of the heart and marked with a pen on the skin. We used local anaesthesia by administering 10 ml of 2% lidocaine (20 mg ml^−1^; Richmond Vet Pharma, Buenos Aires, Argentina) and then did a second surgical scrub with the povidone iodine solution. We made a 3-cm skin incision and loosened the epidermis and subcutis using scissors, creating a pocket to insert the logger with the two electrodes facing the skin (Fig. 1B). To reduce internal movement, a Supralon 3–0 non-absorbable suture was passed through the logger’s anchoring hole, and three additional sutures (dorsal, caudal and ventral relative to the implant) were placed to form a three-sided subcutaneous pocket (Fig. 1C). The incision was closed with 30-mm non-absorbable sutures and treated with topical antiseptic spray (Bactrovet plata AM, König, Buenos Aires Argentina). After the procedure, each animal received 4 ml of an anti-inflammatory [0.5 mg kg^−1^ intramuscularly (I.M.) dexamethasone; Richmond Vet Pharma, Buenos Aires, Argentina] and 10 ml of a long-acting antibiotic (20 mg kg^−1^ I.M. oxytetracycline; Zoetis SRL, Argentina). The complete procedure took 31 min on average.

After implantation, the guanacos were kept in a 6-ha pen with free access to water and natural pasture. They were closely monitored every 48 h during the first week to assess wound healing and general condition. Then, they were observed from a minimum distance of 150 m to minimize disturbance. After 45 days post-implantation, animals were recaptured for logger retrieval following the same methodology described for implantation.

#### Trial 2

Loggers’ sterilization and guanaco handling during surgery were performed as described for trial 1. Based on the experience gained during trial 1 and the manufacturer’s further guidance, we modified the logger model and surgical protocol. In February 2024, we fitted four guanacos with DST micro-HRT loggers (8.3-mm diameter × 25.4-mm length, 3.3 g, Star-Oddi, Iceland; Table 1). This model monitors HR and Tsc and has the same technical specifications as the DST centi-HRT ACT logger. Loggers were programmed using single recording intervals to register baseline physiological data (Supplementary Material  1).

Loggers were implanted subcutaneously in the left intercostal region, approximately 15 cm from the heart (Fig. 1D). This site was possible to use due to the smaller size of the biologgers. In this case, we used general anaesthesia by administering ketamine (5 mg kg^−1^; Richmond Vet Pharma, Buenos Aires, Argentina) and xylazine (0.4 mg kg^−1^; Richmond Vet Pharma, Buenos Aires, Argentina) intramuscularly to decrease handling in the surgical area associated with local anaesthesia. During sedation, the respiratory rate and HR were monitored. A ~2-cm incision was made, and a subcutaneous pocket was created as described above. We also did three stitches around the logger (dorsal, cranial and ventral relative to the implant) to make a three-sided pocket (Fig. 1E and F). The wound was sutured with 30-mm non-absorbable surgical sutures and treated with topical antiseptic (Bactrovet plata AM, König, Buenos Aires, Argentina). Dexamethasone (0.5 mg kg^−1^ IM; Richmond Vet Pharma, Buenos Aires, Argentina) and oxytetracycline (20 mg kg^−1^ IM; Zoetis SRL, Argentina) were administered post-operatively. Yohimbine (0.05 to 0.1 mg kg^−1^ I.V.; Richmond Vet Pharma, Buenos Aires, Argentina) was given as an antagonist agent of xylazine before release. The procedure, including the anaesthesia, took 43 min on average. Guanacos recovered in a sheltered enclosure (3 × 2 m, hay bedding) until fully standing and alert (typically ~5 min post-injection of the antagonist). Then, they were kept in pairs in two 35 × 80-m pens with free access to natural pasture and water. We monitored the animals every 48 h from approximately 35–50 m using binoculars and a spotting scope. Guanacos were recaptured 1 week after the implantation to assess the surgical site and wound healing; animal handling took around 10 min. After 30 days post-implantation, guanacos were recaptured for logger retrieval. Animals were manually restrained, and local anaesthesia was used (5 ml of 2% lidocaine, 20 mg ml^−1^; Richmond Vet Pharma, Buenos Aires, Argentina) together with a post-operative anti-inflammatory (dexamethasone, similar dosage as described above) and a local antibiotic (hetacillin potassium; Boehringer Ingelheim Vetmedica GmbH, United States). A small incision (~2 cm) was made at the top of the logger through which the logger was pushed out. The incision was sutured with 2–0 absorbable surgical sutures. The procedure took ~16 min on average.

### Data processing and analysis

We used HRT Analyser v2.0.0. (Star Oddi, 2025) to inspect and validate ECG traces (Supplementary Material  2). This software enabled us to verify the accuracy of the HR calculation by the algorithm or to determine whether manual annotation was required (Fig. S1). Data validation was performed following Trondrud *et al.* (2021) and Rouyer *et al.* (2023). Values were filtered to 20–176 beats per minute (bpm) based on data validation (Supplementary Material  2), and only records with high electrocardiogram signal quality (QI = 0–1; Star Oddi, 2025) were retained (Fig. S2). For plotting and estimating summary statistics, we excluded data collected during the first 24 h after implantation and the 24 h preceding loggers’ retrieval to avoid measurements influenced by handling.

To characterize the circadian rhythmicity of Tsc and HR, we applied cosinor analyses (Nelson, 1979) with a 24-h period using the cosinor package (v1.2.3, Sachs, 2023) in R software (v4.5.1, R Core Team, 2025). For each individual, we estimated MESOR (Midline Estimation Statistic of Rhythm, representing the average value around which the variable fluctuates), amplitude (the difference between the peak and the mean value of the wave) and acrophase (the time of peak activity). To reduce short-term variability and emphasize physiologically meaningful patterns, Tsc and HR measurements recorded at 10-min intervals were aggregated to hourly means before analysis. Results were expressed as mean ± SE, and a *P* value of <0.05 was considered to be statistically significant.

To evaluate the relationship between Ta and Tsc while accounting for circadian structure, we fitted mixed linear models using the lme4 package (v1.1–37, Bates *et al.*, 2015), including ambient temperature as a continuous fixed effect. The circadian component was incorporated into the model using sinusoidal (sine and cosine) functions; individual identity was used as a random effect to account for repeated measurements within animals. We used the Ta data from the nearest weather station, located around 39 km in a straight line from the Los Peucos farm, which is located in a local airport (40.07° S, 71.13° W; https://aeropuertochapelco.com/institucional/). Model checking was performed using the performance package (v0.15.0; Lüdecke *et al.*, 2021).

## Results

### Implantation methods

#### Trial 1

**Figure 2:**
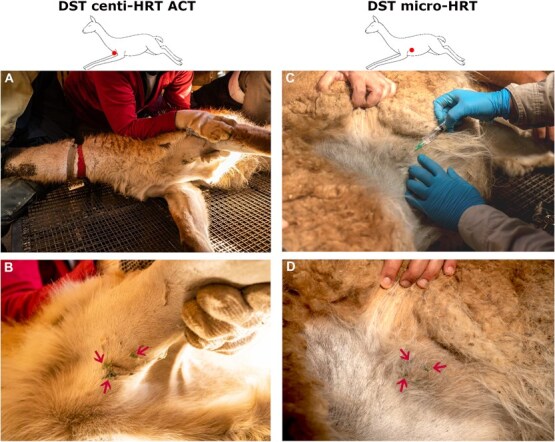
Examples of post-surgical incision condition at the time of recapture for guanacos in trial 1 (A and B) and trial 2 (C and D). Dots in the guanaco silhouettes at the top of the figure indicate loggers’ implantation site. Arrows in photos B and D indicate that the stitches around the logger were still in place.

Implantation of DST centi-HRT ACT loggers resulted in limited haemorrhaging. The surgical site presented loose tissue and flexible skin. During the post-surgical implantation monitoring, we occasionally observed animals scratching the implantation site with their hind legs. Body condition index remained consistent between implantation and recapture for all individuals (BCI = 2–3), indicating that all guanacos were in good physical condition at the end of the study period. Moreover, surgical incisions were fully healed, with no evidence of swelling or infection (Fig. 2A and B). However, none of the implanted loggers was recovered. Subsequent ultrasound examination (SonoScape A5; SonoScape Medical Corp.), combined with manual palpation to assess potential device migration, confirmed that all individuals had expelled the loggers before recapture.

#### Trial 2

Implantation of DST micro-HRT loggers caused less haemorrhaging than in trial 1. The surgical site presented less flexible skin. At the 1-week post-implantation assessment, one individual showed a superficial scratch near the sutured site, and we noticed the implant had rotated caudally; the surgical sites of the other three individuals looked healed. During post-surgical implantation monitoring, no scratching of the implantation site was observed. Approximately 2 weeks after implantation, hair had already grown, and the implantation site was no longer visible. Body condition index remained stable between implantation and recapture (BCI = 2–3), with all individuals in good physical condition at the end of the study period. In contrast to trial 1, all DST micro-HRT loggers were retained and recovered without complication. Surgical incisions healed normally, showing no signs of infection or irritation (Fig. 2C and D), and loggers remained at their original implantation location at retrieval.

### Characterization of HR and subcutaneous body temperature data

Three out of four devices in trial 2 provided continuous, high-quality HR recordings throughout the deployment period, with stable signal quality and no apparent periods of data loss. The implant that had rotated yielded only 28% of records with a QI = 0 or QI =1 and was excluded from data analysis, as data quality declined markedly after the first week post-implantation (from ~55–60% to ~15–25%), likely associated with implant displacement. As a result, the available high-quality data did not adequately represent the full deployment period (Fig. S3). All implants successfully recorded Tsc data. Hourly mean HR and Tsc data for each individual across the full study period are available in Supplementary Material 3 (Fig. S4).

**Figure 3:**
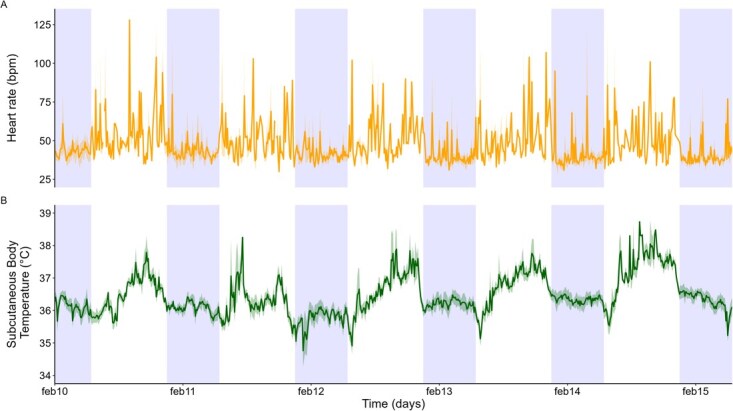
Example of the mean (±SE, depicted with the appropriate colour shading) values of (A) HR and (B) subcutaneous body temperature during 5 days within the study period. Data are from adult non-pregnant female guanacos (*n* = 3 for HR, *n* = 4 for temperature). The variables were measured at 10-min intervals. Shaded areas indicate night phase.

Raw ECG data were available for a total of 964 HR measurements. After validating and filtering the calculated data, 8542 HR recordings (~65% of total recordings) were further analysed for the distribution of HR values. HR ranged between 20 and 170 bpm; around 86% of the data were between 30 and 120 bpm. Mean daily HR ranges differed between day and night across all individuals (Table 2, Fig. 3A). During daytime, mean HR ranges were broad (~99–102 bpm), driven by high maximum values (~124–131 bpm), whereas night-time ranges were consistently reduced (~37–65 bpm) due to lower maximum HRs. Mean minimum HRs were similar during the day and at night (~25–34 bpm), indicating comparable resting values.

**Table 2:** Mean (±SD) daily minimum, maximum and range of HR (bpm) for each individual guanaco, calculated separately for daytime and night-time periods

**Individual ID**	**Period**	**Mean minimum HR (±SD)**	**Mean maximum HR (±SD)**	**Mean HR range (±SD)**	***n***
1	Day	26 (4)	128 (26)	101 (26)	28
1	Night	26 (3)	63 (33)	37 (33)	28
2	Day	26 (4)	124 (34)	98 (34)	28
2	Night	25 (4)	90 (41)	65 (42)	28
3	Day	29 (8)	131 (24)	102 (26)	28
3	Night	34 (4)	88 (26)	54 (27)	28

Values represent averages across days with valid data (*n* = number of days per individual).

**Figure 4:**
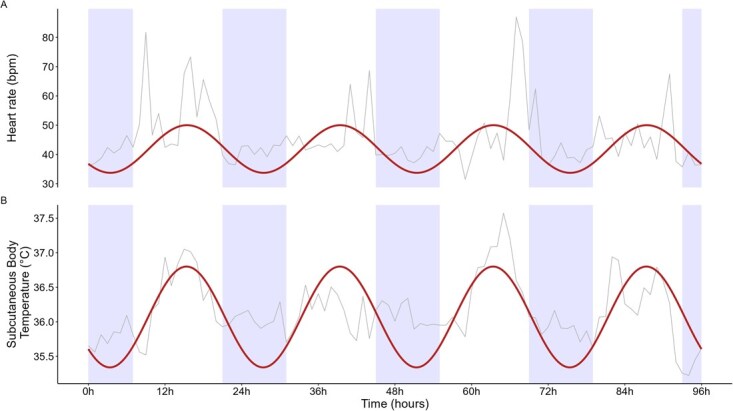
Example of (A) HR and (B) subcutaneous body temperature traces hourly aggregated from one individual guanaco (black lines). The daily fitted cosinor models are shown as a superimposed red trace.

Guanacos exhibited 24-h circadian rhythms in HR (*P* < 0.001, *R*^2^adj = 0.11–0.21), with a mean MESOR of 45 ± 4 bpm, an amplitude of 8 ± 1 bpm and a mean acrophase of 14:42 h (Table S1). The pattern of the HR rhythm for a single representative animal is shown in Fig. 4A.

We collected over 4032 Tsc measurements at 10-min intervals, ranging from 31.84°C to 44.20°C, and a mean ± SD of 36.15 ± 0.91°C. Tsc followed a diurnal rhythm with the lowest value usually just after sunrise and the highest in the afternoon (Fig. 3B). The Tsc difference between daily maximum and Tsc daily minimum varied markedly, ranging from 1.94°C to 6.01°C, and had a mean value of 3.63 ± 0.87°C (Fig. S5).

Guanacos also showed a 24-h circadian rhythm in Tsc (*P* < 0.001, *R*^2^adj = 0.40–0.52), with a mean MESOR of 36.15 ± 0.18°C, an amplitude of 0.90 ± 0.12°C and a mean acrophase of 15:17 h (Table S1, Fig. 4B). The mixed cosinor model showed a positive association between Tsc and Ta (β = 0.091 ± 0.003, *t* = 28.13, *P* < 0.0001), indicating that for every 1°C increase in Ta, subcutaneous body temperature increased by approximately 0.09°C. A significant interaction between ambient temperature and the cosine component was also detected (β = 0.011 ± 0.003, *t* = −3.11, *P* < 0.01). After accounting for ambient temperature, the estimated acrophase of Tsc occurred at 12:20 h.

## Discussion

Continuous physiological monitoring can provide key insight into how wildlife responds to environmental and anthropogenic challenges, yet obtaining long-term physiological data from large free-ranging mammals remains technically challenging. In this study, we evaluated the feasibility of subcutaneous physiological biologging in guanacos by comparing two implantation trials that differed in biologger model and surgical approach. The contrasting retention outcomes between trials highlight the importance of species-specific pilot validation before field deployment. While the simultaneous variation in multiple methodological factors prevents definitive attribution of retention success to a single variable, the smaller biologger implanted in the intercostal region achieved complete retention. These loggers yielded the first continuous records of HR and subcutaneous body temperature in guanacos. The data revealed pronounced daily physiological rhythms and environmentally responsive thermal patterns, demonstrating the potential of implantable biologgers to support conservation physiology research in large ungulates.

### Implantation methods

The two implantation trials yielded markedly different results. The larger implant showed a high acute rejection rate in guanacos, with all devices expelled within 4 weeks, despite the absence of apparent surgical complications. Notably, guanacos showed rapid wound healing, as no external evidence of implant rejection or expulsion was observed at the time of retrieval. We hypothesize that implantation in a region characterized by greater tissue mobility, higher vascularization and looser connective structure may have promoted a pronounced inflammatory response and expulsion of the foreign body.

Retention outcomes reported in other species similarly indicate that device size, implantation site and species-specific tissue responses strongly influence success. For example, Fuchs *et al.* (2019) reported early post-surgical rejection (days 12–20) in three of the eight lambs that lost implanted loggers, likely associated with wound infection and compromised health status. Laske *et al.* (2018) reported an 83% (five out of six devices) foreign body rejection rate within 90 days in black bears implanted with larger cardiac monitors, with reduced rejection rates following the use of smaller devices. In this species, decreasing logger size appeared to shift responses from acute inflammation towards long-term chronic responses (Laske *et al.*, 2018). Consistent with these findings, the smaller implant used in our study showed substantially improved retention, minimal tissue response and no evidence of migration during the deployment period. Together, these results suggest that implant size and implantation site may represent important factors influencing device retention in guanacos, and likely in other large mammals. However, because the two trials involved concurrent changes in device specifications, implantation site and anaesthetic protocols, the specific drivers of improved device retention cannot be isolated. Importantly, the rejection of the larger biologger model, despite technically successful surgeries, underscores that device retention cannot be assumed *a priori* and requires species-specific validation to safeguard data reliability and animal welfare in conservation physiology research.

Based on the comparison between trials, several practical considerations emerge from this study. First, smaller and lighter implants, such as the DST micro-HRT loggers, may represent promising models for future applications in guanacos and potentially other large mammals. Second, the left intercostal region appears suitable for the implantation of small loggers, whereas larger devices may be less appropriate for this site due to their greater size. Third, although local anaesthesia reduces handling time, sedation may be advantageous in large mammals like guanacos, as it minimises manipulation at the implantation site and improves safety for both animals and handlers. Recovery was also rapid (approximately 5 min) and secure for the animals. Finally, intensive post-implantation monitoring beyond the initial 2 weeks may be unnecessary, as hair regrowth quickly obscures the implantation site. Moreover, the complete wound healing observed in trial 1 suggests that implant expulsion likely occurred within the first 15 days post-implantation.

### Characterization of HR and subcutaneous body temperature data

DST micro-HRT loggers yielded the first continuous records of HR and Tsc in guanacos and illustrated the added value of continuous physiological monitoring relative to ‘snapshot’ measurements. Continuous monitoring allowed the characterization of daily physiological rhythms and baseline reference ranges. Minimum HR values in our study were lower than those published in captive (Zapata *et al.*, 2004) and wild guanacos (Carmanchahi *et al.*, 2011). However, these studies report HR data during handling over short time periods (i.e. from a single measurement to around a 4-h period). Resting HR values in our study are closer to those reported for Old World camelids, which ranges typically between 35 and 50 bpm (*Camelus dromedarius,*  Tefera, 2004). Moreover, HR patterns showed reduced variability at night, consistent with prolonged resting behaviour, such as lying down, resting or sleeping. Conversely, broader daytime ranges reflect increased activity, typical of diurnal mammals (de Lamo *et al.*, 1998; Leimgruber *et al.*, 2023), including behaviours like walking, foraging or running, which require increased oxygen consumption for muscle activation (Niccolai *et al.*, 2024).

Mean Tsc values were comparable to those reported for other South American camelids (SAC; Niehaus, 2022). Subcutaneous body temperature exhibited a clear daily rhythm that was influenced by Ta, with environmental conditions contributing to the timing and magnitude of observed temperature fluctuations. After accounting for Ta, the timing of the temperature peak shifted earlier compared to raw observations, suggesting that environmental heat accumulation during the afternoon delays the timing of the maximum recorded temperature. Although preliminary, our data showed pronounced differences between daily minimum and maximum Tsc values resembling patterns reported in related species such as the llama (*Lama glama*; Riek *et al.*, 2019). These patterns may be consistent with heterothermy as an energy-saving strategy under challenging environmental conditions, such as energy or water limitation. More broadly, they relate to an open debate in ecophysiology regarding whether such fluctuations represent ‘adaptive heterothermy’—an actively implemented strategy to preserve resources—or a ‘sub-optimal response’, which reflects a thermoregulatory failure when an animal’s capacity is exceeded by environmental or nutritional stress (Hetem *et al.*, 2016). As our study was not conducted during a period of food shortage and guanacos had free access to water, it remains unclear whether these preliminary findings reflect a regulated adaptive benefit or a functional compromise to environmental challenges. Further research, including a larger sample size under more variable conditions, is required to disentangle these physiological drivers.

### Limitations and further research

This study has some methodological limitations that should be considered when interpreting the results. The sample size was small, and implantations were conducted under captive conditions, which may have influenced activity patterns and physiological variability compared to free-ranging animals. Deployment duration was limited, preventing assessment of long-term implant retention, chronic tissue responses and seasonal effects. Moreover, while the results suggest that device size and implantation site are influential, the simultaneous variation in multiple factors prevents a definitive attribution of retention success to a single variable.

Although smaller implants improved retention, their reduced inter-electrode distance likely decreases signal amplitude and increases susceptibility to movement-related noise (Puurtinen *et al.*, 2009). This may partly explain reduced signal quality during active periods. In this sense, intermediate-sized devices balancing retention and signal robustness merit further evaluation.

Building on this framework, future work should increase sample sizes, extend deployment duration and test the protocol in free-ranging guanacos. Furthermore, integrating physiological biologging with movement and activity data (e.g. GPS or accelerometry) will enable researchers to address key conservation questions, such as quantifying physiological responses to human disturbance (e.g. competition with livestock, hunting), assessing energetic and thermoregulatory costs under fluctuating forage and water availability and identifying periods of heightened stress during harsh winters or drought. Given the inherently low energy expenditure of SAC (Dittmann *et al.*, 2014), such data are critical to assess how anthropogenic stressors may compromise metabolic efficiency. They will also help determine whether observed heterothermy reflects an adaptive strategy or a physiological constraint under increasing environmental challenges. Together, these approaches will provide mechanistic insight into how environmental and anthropogenic factors influence individual performance and, ultimately, population dynamics, supporting evidence-based conservation and management.

## Conclusions

This study presents the first assessment of HR and subcutaneous body temperature biologging in guanacos, demonstrating the feasibility of implantable physiological monitoring in this species. Retained implants yielded reliable, high-resolution cardiac and thermal data, revealing daily physiological rhythms and environmentally responsive temperature patterns. Although further validation is required, these findings highlight the value of pilot studies for refining implantation protocols, assessing welfare outcomes and improving biologger performance before broader application in free-ranging populations. Overall, this work provides practical guidance to support reproducible, welfare-oriented applications of implantable biologging in conservation physiology.

## Supplementary Material

Web_Material_coag048

## Data Availability

The data underlying this article will be shared on reasonable request to the corresponding author.
