# Supplementary material for: Assessing the potential of subcutaneous biologging tools for physiological monitoring in a large terrestrial mammal, the Guanaco (Lama guanicoe)
Source: Conserv Physiol. 2026 Jul 15;14(1):coag048. doi: 10.1093/conphys/coag048 (PMC13368605; doi:10.1093/conphys/coag048)
Supplement: Web_Material_coag048 [file web_material_coag048.zip › Panebianco et al_supplementary material_v3_R2_clean.pdf]

Assessing the potential of subcutaneous biologging tools for physiological monitoring in a large terrestrial mammal, the Guanaco (*Lama guanicoe*)

Panebianco, A[1*]; Rago, M.V.[2]; Gregorio, P.[1]; Peña Lodis, F.[1, 3]; Owen, M.[4]; Pomponio, V.[1]; Pompei, M.P.[5]; Anello, M.[1]; Schroeder, N.M.[1,3,6]; Ovejero, R.[1,7]; Carmanchahi P.[1]

[1] Grupo de Investigación de Eco-Fisiología de Fauna Silvestre (GIEFAS), Instituto de Investigaciones en Biodiversidad y Medio Ambiente, Consejo Nacional de Investigaciones Científicas y Técnicas, Centro Regional Universitario San Martín de los Andes, Universidad Nacional del Comahue. Pasaje de la Paz 235, San Martín de los Andes, Neuquén, Argentina.

[2] Instituto de Investigaciones en Biodiversidad y Medio Ambiente, Consejo Nacional de Investigaciones Científicas y Técnicas, Sede Junín de los Andes, Ruta N° 61 km 3, Junín de los Andes, Neuquén, Argentina.

[3] Witral. Red de Investigaciones en conservación y manejo de vida silvestre en sistemas socio-ecológicos, Instituto Argentino de Investigaciones de las Zonas Áridas, Consejo Nacional de Investigaciones Científicas y Técnicas. Av. Ruiz Leal s/n, Parque General San Martín, Mendoza, Argentina.

[4] San Diego Zoo Wildlife Alliance,15600 San Pasqual Valley Rd., Escondido, CA, United States

[5] Grupo de Investigación en Arqueología Andina, Consejo Nacional de Investigaciones Científicas y Técnicas, Universidad Nacional de Tucumán. Miguel Lillo 205, San Miguel de Tucumán, Tucumán, Argentina.

[6]Facultad de Ciencias Agrarias, Universidad Nacional de Cuyo, Almirante Brown 500, Luján de Cuyo, Mendoza, Argentina.

[7]Instituto de Biodiversidad Neotropical, IBN-CONICET-UNT; CCT NOA SUR. Cúpulas Horco Molle. Yerba Buena, Tucumán, Argentina.

Supplementary material 1: Biologgers programming

We used Mercury software V6.8 (Star-Oddi, Iceland) to program the loggers and then download the data.

Trial 1

DST centi-HRT ACT loggers were programmed using multiple recording intervals. During drone approach (i.e., three times a week for four weeks), activity, temperature and heart rate were recorded at 30-s intervals; heart rate data were recorded at a frequency of 200Hz. ECG was recorded at the same interval (30 seconds) for a subset of the recordings (100 out of 1200) for later validation of heart rate readings. The rest of the days, data was recorded at 40-minute intervals.

Trial 2

DST micro-HRT loggers were programmed using single recording intervals. Heart rate and temperature data were recorded at 10-minute intervals; heart rate data were recorded at a frequency of 150 Hz. ECG was recorded every three hours for later validation of heart rate readings.

Supplementary material 2: Validation and filtering of heart rate data

We filtered HR data following a validation approach similar to that described by Trondrud *et al.*, (2021) and Rouyer *et al.*, (2023). We visually inspected and validated 964 ECG traces using HRT Analyzer software v2.0.0 from Star-Oddi, recorded with the DST micro-HRT loggers during trial 2. The software algorithm calculates the heart rate (HR), Quality Imdex (QI) and estimates heart rate variability (HRV) based on raw ECG data. It assigns peaks to the biggest slope of the QRS waveforms, where the most significant difference is between two points. Then, we validated each record to check if HR was being calculated correctly by the software algorithm or if manual annotation was needed, for instance, when QRS peaks were selected inaccurately (Fig. S1). This was done by pressing each valid peak of the QRS waveform in the ECG. Each ECG trace is 4 seconds long, sampled at 150Hz. The ECG trace goes through on-board processing for heart rate and a quality estimation (QI).

Figure S2 shows the correlation between HR values calculated by the logger's on-board algorithm and independently validated HR estimates for each of the quality indices (QI 0–3). We established the lower HR threshold (20 bpm) based on published heart rate ranges reported for other free-ranging ungulate species (Trondrud *et al.*, 2021). Across quality levels, logger-derived HR estimates showed a marked decline in reliability at values exceeding 176 bpm, including recordings classified as QI 0 (Fig. S2). In particular, HR values graded as QI 3 showed poor correlation with validated measurements, and we therefore excluded them from subsequent analyses. Finally, based on R-squared values (Fig. S2), we retained only recordings with QI = 0 and QI = 1 that fell between 20 and 176 bpm, and excluded measurements graded as QI = 2 or QI = 3.

A

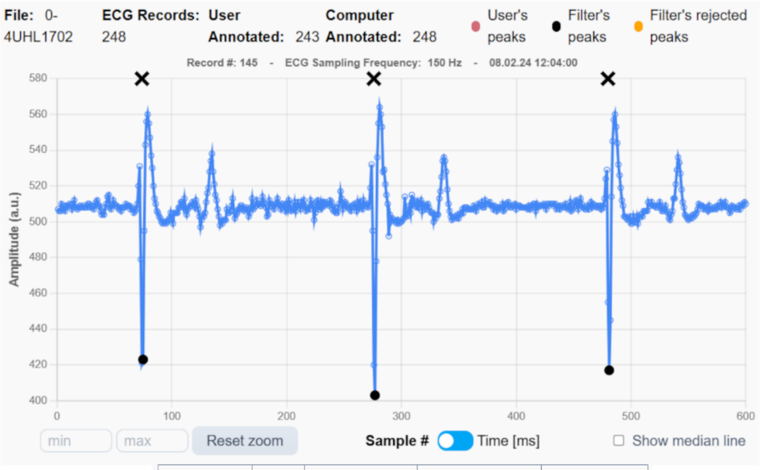

	BPM	HRV (SDNN)	HRV (RMSSD)	QI
User				– 0 +
Computer	44	9.43 ms	13.33 ms	0
Logger	44			0

B

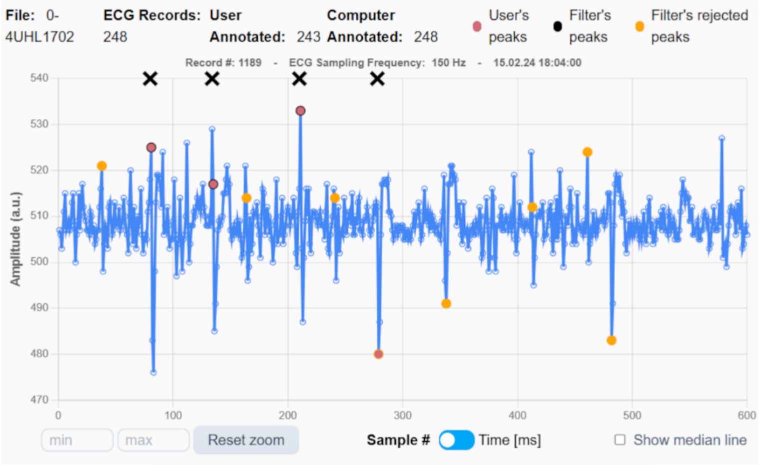

	BPM	HRV (SDNN)	HRV (RMSSD)	QI
User	136	74.24 ms	110.35 ms	– 0 +
Computer	138	103.71 ms	146.67 ms	2
Logger	138			2

Figure S1: Examples of ECG processed with the Analyzer software v2.0.0. The computer algorithm detects QRS waveforms and assigns peaks to the biggest slopes (black dots and crosses). The software calculates heart rate variability (HRV) as SDNN (standard deviation of normal to normal R-R intervals) and RMSSD (root mean square of successive differences between normal heartbeats), shown in the tables. (A) Example of an ECG calculated correctly by the software algorithm. Usually, these records had QI= 0 and QI= 1 and corresponded to resting heart rates. (B) Example of an ECG where manual annotation was needed (red dots). Usually, these records had QI= 2 and QI= 3 and were during the active period.

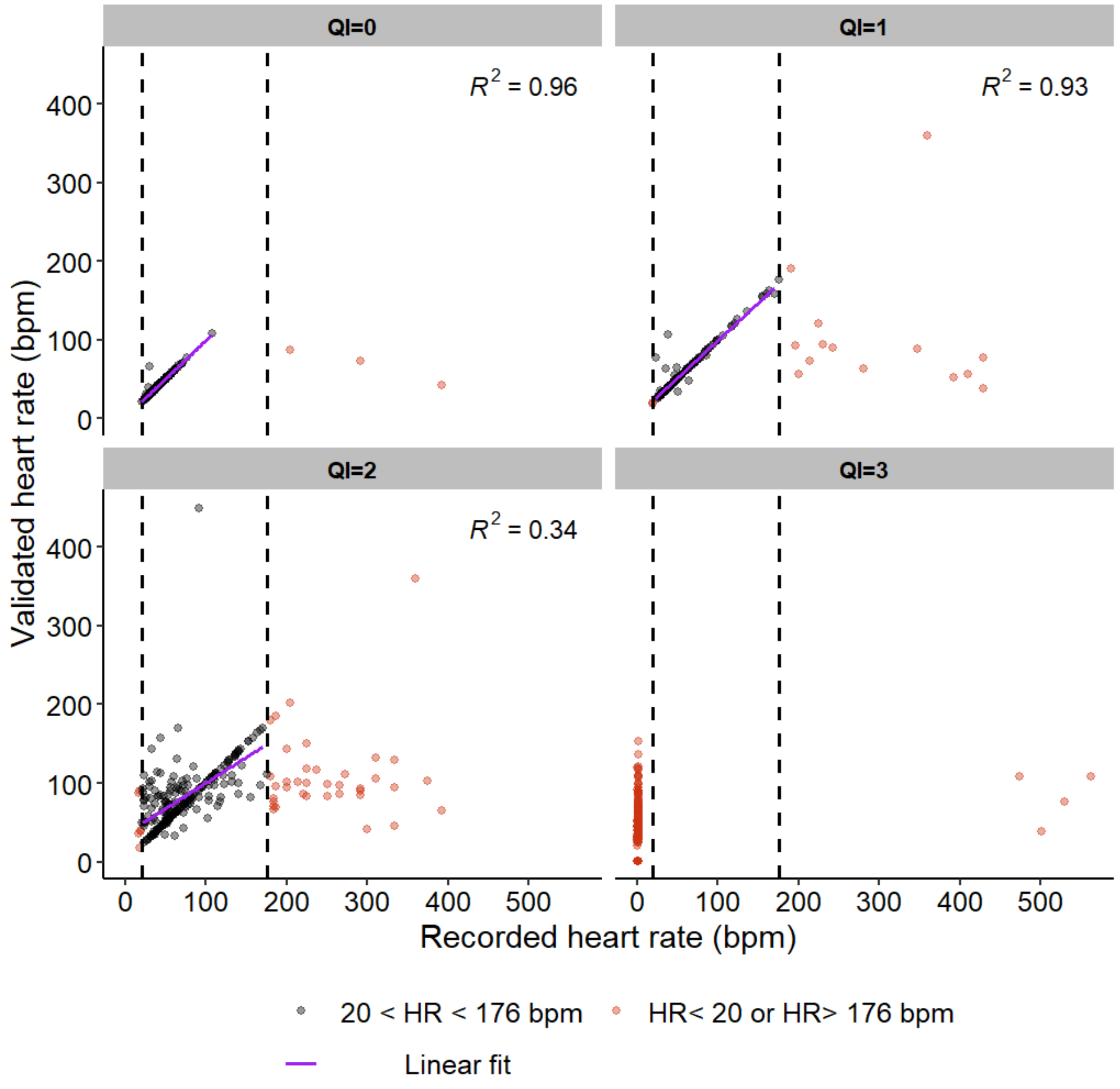

Figure S2: Correlation between recorded heart rate by the DST micro-HRT loggers during trial 2 and manually validated heart rates from raw ECG signals, grouped by quality index. Dashed lines indicate the minimum and maximum heart rate values considered for data filtering (20 and 176 bpm). The purple solid line represents the fitted linear correlation between the recorded and validated heart rates within the 20–176 bpm range, with corresponding R^2 values shown.

Supplementary material 3: Logger data

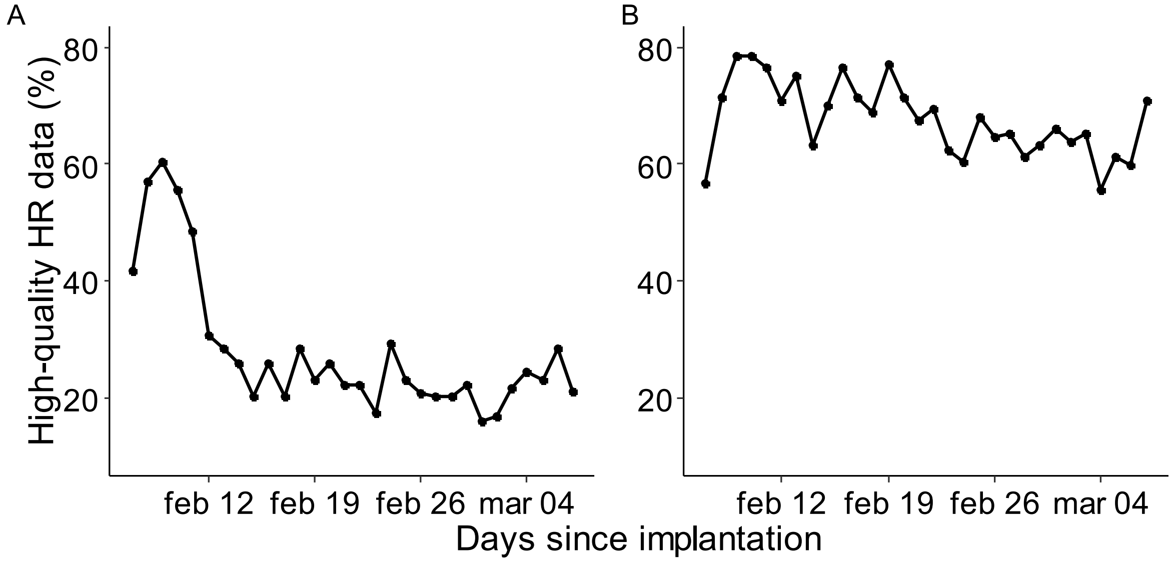

Figure S3: Percentage of high-quality heart rate records (QI = 0–1) across days since implantation for A) the rotated implant, which had with 28% of records with a QI = 0 or QI =1; and B) an implant with 68% of records with a QI = 0 or QI =1 that had not rotated. Data quality declined sharply after 12 February (from ~55–60% to ~15–25%), indicating temporal bias likely associated with implant displacement, which was observed at the one-week post-implantation check (15 February).

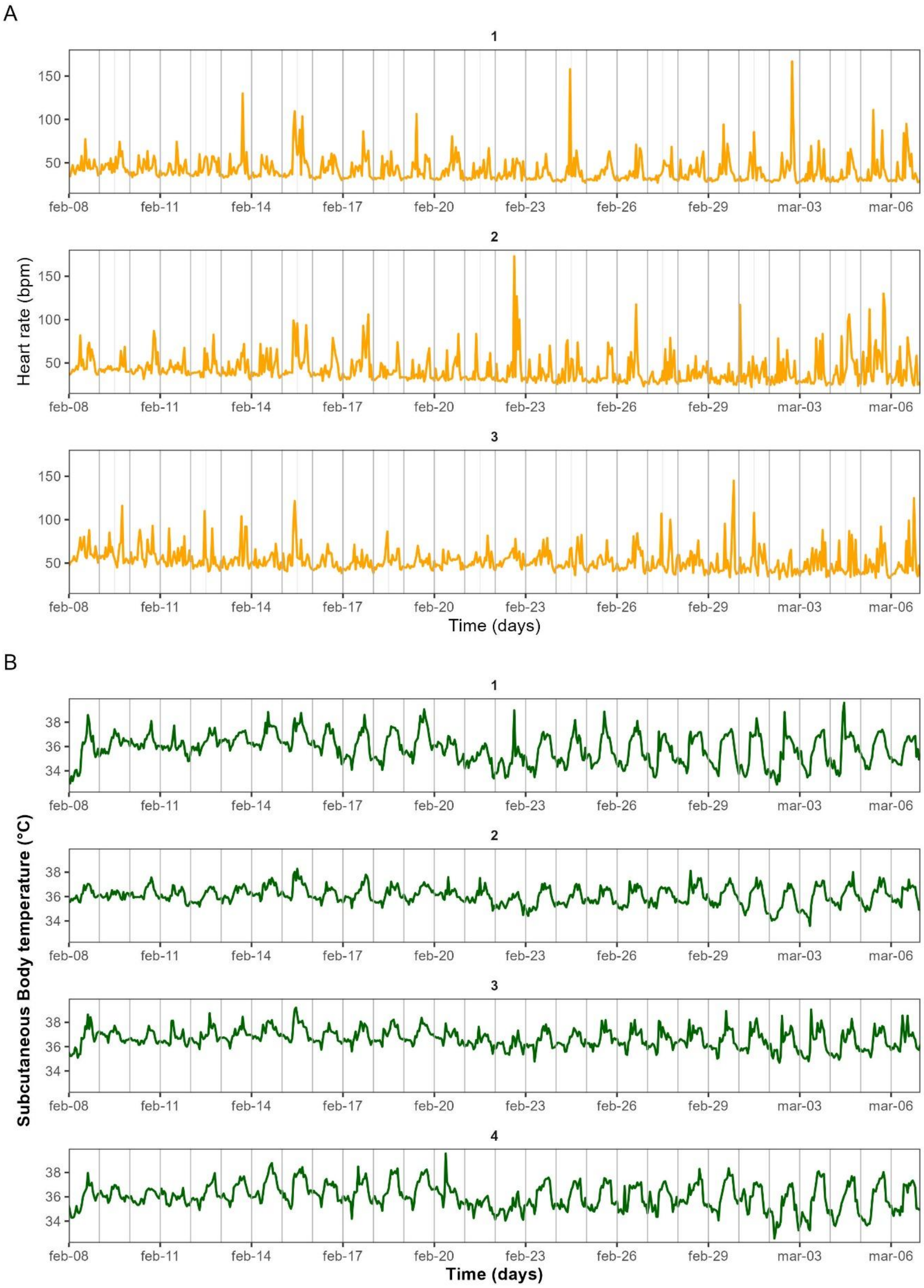

Figure S4: Hourly mean heart rate (A) and body temperature (B) of adult female guanacos monitored using DST micro-HRT loggers. Data represent hourly averages calculated from 10-min recordings

after excluding measurements obtained during the first 24 h following implantation and the last 24 h before retrieval. Panels with numbers 1-4 correspond to individual animals. The vertical grey lines separate different days. For heart rate, three individuals are plotted as one logger had unreliable heart rate data.

Cosinor analyses

Table S1: Individual cosinor rhythms for heart rate (beats per minute) and subcutaneous body temperature (°C) in adult female guanacos. The table shows the values of Midline Estimation Statistic of Rhythm (MESOR), amplitude (the difference between the peak and the mean value of a wave), and acrophase (the time of peak activity, expressed in hh:mm) of the cosine curve of a 24-h activity rhythm.

	**Animal ID**	**Mesor**	**Amplitude**	**Acrophase**	**R^2 adj**
**Heart rate**	1	41	9	14:21	0.21
	2	41	8	15:26	0.11
	3	53	7	14:18	0.13
**Subcutaneous body temperature**	1	35.84	4.00	15:16	0.48
	2	36.07	0.73	15:22	0.52
	3	36.67	0.69	14:58	0.40
	4	36.02	1.01	15:29	0.44

Correlation between ambient temperature and Subcutaneous body temperature

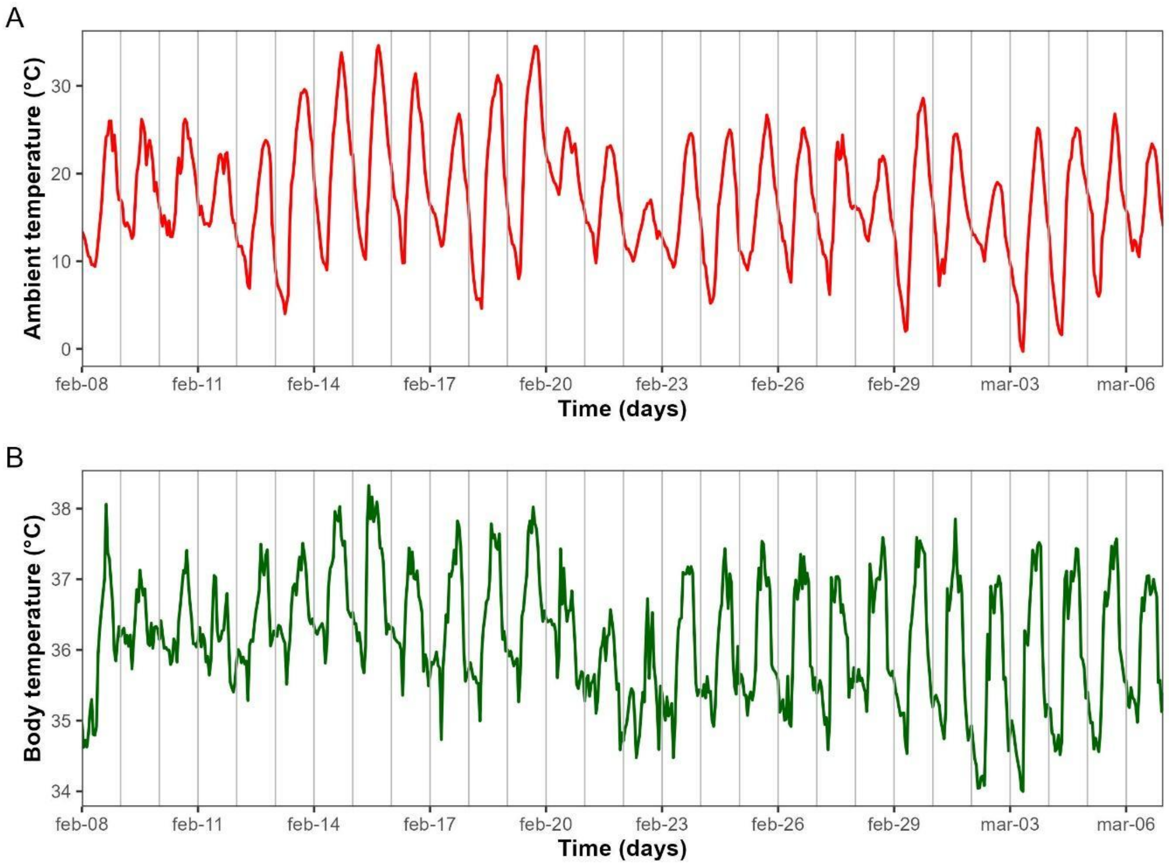

Figure S5: Daily rhythm of the (A) subcutaneous body temperature, and (B) ambient temperature during the sampling period. Subcutaneous body temperature data are hourly means from four adult non-pregnant guanacos (n = 2688). The different days are separated by vertical grey lines.

References

Bates D, Maechler M, Bolker B, Walker S, Haubo R, Christensen B, Singmann H, Dai B, Scheipl F, Grothendieck G, *et al.* (2018) Linear Mixed-Effects Models using “Eigen” and S4.

Lüdecke D, Ben-Shachar MS, Patil I, Waggoner P, Makowski D (2021) performance: An R Package for Assessment, Comparison and Testing of Statistical Models. *Journal of Open Source Software* 6: 3139.

Rouyer T, Bonhommeau S, Bernard S, Kerzerho V, Derridj O, Bjarnason Á, Allal H, Steffensen JF, Deguara S, Wendling B, *et al.* (2023) A novel protocol for rapid deployment of heart rate data storage tags in Atlantic bluefin tuna reveals cardiac responses to temperature and feeding. *Journal of Fish Biology* 106: 1305–1315.

Trondrud LM, Pigeon G, Albon S, Arnold W, Evans AL, Irvine RJ, Król E, Ropstad E, Stien A, Veiberg V, *et al.* (2021) Determinants of heart rate in Svalbard reindeer reveal mechanisms of

seasonal energy management. *Philosophical Transactions of the Royal Society B: Biological Sciences* 376: 20200215.
